# BRCAness Profile of Sporadic Ovarian Cancer Predicts Disease Recurrence

**DOI:** 10.1371/journal.pone.0030042

**Published:** 2012-01-11

**Authors:** Weiya Z. Wysham, Paulette Mhawech-Fauceglia, Hong Li, Laura Hays, Suzanna Syriac, Tijana Skrepnik, Jay Wright, Nupur Pande, Maureen Hoatlin, Tanja Pejovic

**Affiliations:** 1 Department of Obstetrics and Gynecology, Oregon Health & Science University, Portland, Oregon, United States of America; 2 Knight Cancer Institute, Portland, Oregon, United States of America; 3 Department of Pathology, Roswell Park Cancer Institute, Buffalo, New York, United States of America; 4 Department of Biostatistics, Oregon Health & Science University, Knight Cancer Institute, Portland, Oregon, United States of America; 5 University of Arizona, Tucson, Arizona, United States of America; 6 Department of Biochemistry and Molecular Biology, Oregon Health & Science University, Portland, Oregon, United States of America; Sun Yat-sen University Medical School, China

## Abstract

**Background:**

The consequences of defective homologous recombination (HR) are not understood in sporadic ovarian cancer, nor have the potential role of HR proteins other than BRCA1 and BRCA2 been clearly defined. However, it is clear that defects in HR and other DNA repair pathways are important to the effectiveness of current therapies. We hypothesize that a subset of sporadic ovarian carcinomas may harbor anomalies in HR pathways, and that a BRCAness profile (defects in HR or other DNA repair pathways) could influence response rate and survival after treatment with platinum drugs. Clinical availability of a BRCAness profile in patients and/or tumors should improve treatment outcomes.

**Objective:**

To define the BRCAness profile of sporadic ovarian carcinoma and determine whether BRCA1, PARP, FANCD2, PTEN, H2AX, ATM, and P53 protein expression correlates with response to treatment, disease recurrence, and recurrence-free survival.

**Materials and Methods:**

Protein microarray analysis of ovarian cancer tissue was used to determine protein expression levels for defined DNA repair proteins. Correlation with clinical and pathologic parameters in 186 patients with advanced stage III–IV and grade 3 ovarian cancer was analyzed using Chi square, Kaplan-Meier method, Cox proportional hazard model, and cumulative incidence function.

**Results:**

High PARP, FANCD2 and BRCA1 expressions were significantly correlated with each other; however, elevated p53 expression was associated only with high PARP and FANCD2. Of all patients, 9% recurred within the first year. Among early recurring patients, 41% had high levels of PARP, FANCD2 and P53, compared to 19.5% of patients without early recurrence (p = 0.04). Women with high levels of PARP, FANCD2 and/or P53 had first year cumulative cancer incidence of 17% compared with 7% for the other groups (P = 0.03).

**Conclusions:**

Patients with concomitantly high levels of PARP, FANCD2 and P53 protein expression are at increased risk of early ovarian cancer recurrence and platinum resistance.

## Introduction

Ovarian cancer is the second most common gynecologic malignancy and the most common cause of death among women with a gynecologic cancer. About 21,000 ovarian cancer cases are diagnosed annually, and about 14,000 deaths from ovarian cancer occur each year [1]. Although significant improvements have been made over the past several decades to extend median life span after ovarian cancer diagnosis, less than 30% of patients can be cured with optimal surgical cytoreduction and standard adjuvant chemotherapy with a platinum-containing agent, and long term survival has not improved [2].

Patients with mutations of BRCA1 or BRCA2, genes involved in the repair of double-stranded DNA breaks by homologous recombination (HR), have increased sensitivity to platinum chemotherapy, thus improving their overall survival outcome [3]–[5]. Additionally, cells with pre-existing defects in the HR pathway (BRCA 1 or BRCA 2 mutation) can be targeted by a second hit in the form of an inhibition of the base excision repair (BER) pathway which leads to cell death. This concept of synthetic lethality – the combination of two genetic alterations, which on their own are non-lethal, but together result in a lethal phenotype – led to interest in inhibitors of BER pathways. One such BER inhibitor is the poly-ADP ribose polymerase (PARP) inhibitor, which makes HR deficient cells particularly sensitive to chemotherapy-induced DNA injury [6]–[8]. While normal cells can repair the damage and survive, the BRCA-deficient cells cannot activate the HR system and therefore they die (6). PARP inhibitors have demonstrated promising results in patients with BRCA1- or BRCA2-positive ovarian cancer [9]–[11].

While only 10–15% of women with ovarian cancer have germline BRCA1 or BRCA2 mutations, recent data suggest that sporadic ovarian cancers can harbor acquired genetic and epigenetic defects in BRCA and in other DNA repair genes and proteins, such as PTEN, RAD51, and Fanconi anemia (FA) genes [7], that may contribute to the “BRCAness profile”. Given the shared role that BRCA1 and BRCA2 have with other DNA repair genes, defects in these other DNA repair proteins alone could influence response to treatment, recurrence rates, and overall survival and increase sensitivity to PARP inhibitors. The challenge is to define BRCAness profiles in sporadic ovarian cancer and correlate this with clinical outcome.

Thus, the objective of this study is to define the BRCAness profile of sporadic ovarian cancer. Specifically we aimed to determine whether the expression of DNA repair proteins, including PARP, FANCD2, BRCA1, PTEN, H2AX, ATM, as well as p53, correlated with response to treatment, recurrence rate, and survival in ovarian cancer.

## Materials and Methods

### Ethics Statement

All patients underwent ovarian cancer staging or debulking surgery at the Roswell Park Cancer Institute in Buffalo, New York. All pathology specimens were collected and reviewed in our institution, and tumors were classified according to WHO criteria [12]. The medical records of the patients were retrospectively reviewed under an approved Roswell Park Cancer Institute Institutional Review Board protocol that requires written patient consent. The review included out- and inpatient treatment, including surgery and chemotherapy. Overall survival and time to progression were determined, each measured from the time of diagnosis (at initial surgery). Progression was defined as objective evidence of recurrence. The duration of overall survival was the interval between diagnosis and death. Observation time was the interval between diagnosis and last contact (death or last follow-up). Data were censored at the last follow-up for patients with no evidence of recurrence, progression, or death.

### Antibodies

Six commercially available primary monoclonal antibodies were used, specific for ATM (Abcam, Boston, MA), BRCA1 (Biocare lab, Concord, CA), FANCD2 (Epitomics, San Francisco, CA), PARP (Abcam), p53 (Novacastra, Lica Microsystems, Buffalo Grove, IL), PTEN (Millipore, Billerica, MA), and a polyclonal antibody to H2AX (Bethyl Laboratories, Montgomery, TX) (Table 1). Detection was performed using the avidin-biotin-peroxidase complex method (LASB-kit, Dakocytomation, Glostrup, Denmark).

**Table 1:** Immunocytochemistry.

Antibody	Product	Cat. #	Dilution	Antigen retrieval	Incubation
ATM	Abcam	Ab78	1/50	TRS 40 min steamer	1 hour
FANCD2	Epitomics	S2909	1/100	Citrate buffer 20 min microwave	1 hour
HA2AX	Bethyl Lab	A300-082A	1/100	Citrate buffer 20 min microwave	1 hour
P53	Novacastra	PA0057	1/50	Citrate buffer 20 min steamer	1 hour
PARP	Abcam	Ab110915	1/25	Citrate buffer 20 min steamer	1 hour
PTEN	Millipore	04-035	1/100	Citrate buffer 20 min microwave	2 hours
BRCA1	Biocare	CM345A,C	1/1000	Citrate buffer 20 min, room temperature	Overnight

### Tissue MicroArray Preparation

Paraffin-embedded tissues from 202 patients with ovarian cancer were used to construct tissue microarrays as described previously by Kononen et al. [13]. Briefly, after carefully choosing the morphologically representative region from the hematoxylin-eosin (HE) section, 0.6 mm cores were punched from the individual paraffin-embedded blocks (donor blocks), and transferred to the receiver paraffin-embedded block (receiver block). To overcome tumor heterogeneity, core biopsies were performed from three different areas of each tumor. One section was stained with H&E to confirm the presence of the tumor by light microscopy.

Sections were incubated with antibodies to ATM, BRCA1, FANCD2, PTEN, H2AX, PARP, and p53 (Table 1). The biotin-free HRP enzyme-labeled polymer of the Envision Plus Detection System (Dakocytomation) was used as a secondary reagent. The diaminobenzidine complex was used as a chromogen. As a positive control, endometrioid adenocarcinoma of the endometrium was used for PTEN, ovarian serous carcinoma for p53, breast carcinoma for PARP, ATM and BRCA1, normal tonsil for FANCD2, and squamous cell carcinoma for H2AX. For negative controls, a normal goat serum was used instead of the primary antibody. The extent of immunochemical reactivity was graded based on intensity as follows: 0 (background), 1+ (light), 2+ (moderate), 3+ (strong). Negative control slides omitting the primary antibody were included in all assays.

### Statistical Analyses

Statistical analyses were performed using SAS statistical software (version 9.2; SAS Institute Inc, Cary, NC). The seven DNA repair proteins (BRCAness profile) were measured at baseline and were grouped as negative (no or light staining) and positive (moderate or strong staining) based on staining intensity. The associations between baseline cancer status (grade, stage, histology type, and presence of residual tumor) and BRCAness profile and the associations among p53, PARP, FANCD2 and BRCA1 were examined using the Chi square and Cochran Armitage trend tests.

Where possible, time from diagnosis to progression/recurrence within the first 3 years was examined. Patients who died within 3 years were censored at the time of death, and patients who had recurrence after 3 years or who did not have recurrence were also censored at 3 years. The correlation of each protein staining intensity and concurrence of multiple high levels of proteins with 3-year recurrence-free survival were evaluated using the Kaplan-Meier (KM) method. Results from the KM method were then verified by cumulative incidence rates with the event defined as recurrence and the competing risk defined as non-recurrence death using the cumulative incidence function [14]. Age and cancer status adjusted hazard ratio of 3-year recurrence was estimated using the Cox proportional hazard model. Association between concurrence of multiple high levels of proteins and recurrence status at 6 months and 1 year were also explored. All reported p-values are two-sided, and a value of p<0.05 was considered statistically significant.

## Results

Patient characteristics at baseline are summarized in Table 2. A total of 186 of 202 patients were studied. The mean age of the study population was 62 years (range 33 to 89) and the median duration of follow-up for recurrence was 22 months (range 20 days to 3 years). As expected, the majority of the patients had poorly differentiated, grade 3 cancers (161/186, 86.6%), stage III or IV disease (174/186, 93.5%), and serous histology (164/186, 88.2%). At surgery 59 patients had no residual disease while 125 patients had at least minimal residual disease. All patients received platinum-based first line chemotherapy. The median estimated overall survival for all patients was 40.7 months (95% CI, 36.8–45.0 months). The survivorship between patients with absence of residual tumor (58.1%) and presence of residual tumor (59.7%) were similar.

**Table 2:** Patients' Baseline Characteristics.

Characteristics	Patients
	No. (%)
**No. Patients**	186
**Age (33 to 89 years old)**	
Mean ± Std	62.0±11.8
<50	30 (16.1)
50 to 59	50 (26.9)
60 to 79	51 (27.4)
> = 70	55 (29.6)
**Cancer status at diagnosis**	
Histology Type	
Serous	164 (88.2)
Clear cell	10 (5.4)
Endometrioid	8 (4.3)
Mucinous	4 (2.1)
Stage	
I	4 (2.2)
II	8 (4.3)
III	149 (80.1)
IV	25 (13.4)
Grade	
1	7 (3.8)
2	18 (9.7)
3	161 (86.6)
**Residual tumor at surgery**	
Absent	59 (32.1)
Present	125 (67.9)

### Expression of DNA repair proteins in epithelial ovarian cancer

**Figure 1:**
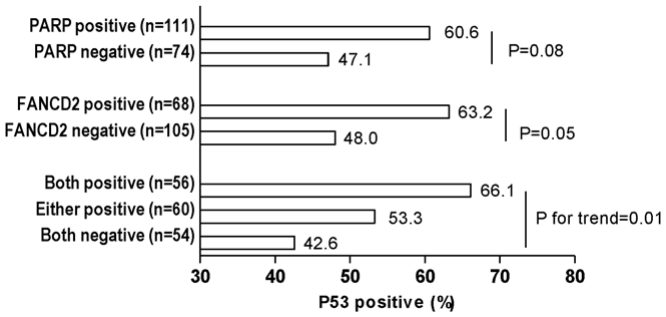
Associations of PARP and/or FANCD2 with P53. Association between PARP, FANCD2, and P53. Patients with positive PARP or positive FANCD2 were more likely to have positive P53. Patients positive for both PARP and FANCD2 were statistically more likely to stain positive for P53.

The protein expression of ATM, FANCD2, PARP, PTEN, H2AX, BRCA1 and p53 in epithelial ovarian tumor specimens was investigated by immunocytochemistry and their associations with ovarian cancer recurrence in 3 years are summarized in Table 3. PARP was positive in 111 (60%) of the cases, FANCD2 was positive in 68 cases (39%), H2AX was positive in 59% and p53 in 55% of the cases. In contrast, most tumors were negative for BRCA1 (87%), PTEN (in 89%) and ATM (92%). Although not statistically significant, KM estimated 3-year recurrence-free survivals for patients with high PARP (56.5% vs. 67.5%) and high FANCD2 (55.5% vs. 66.9%) were more than 10% lower than their low staining counterparts. Patients with high levels of PARP and/or FANCD2 were more likely to also have high p53, 66% in both positive, 53% in either positive, and 43% in both negative were found have positive p53 (p for trend = 0.01, Figure 1). Overall, expression of either FANCD2 or PARP was observed in 35% of the specimens. Co-expression of PARP and FANCD2 was demonstrated in 56 of 173 (32%) specimens. High expression of PARP, FANCD2 and BRCA1 were significantly associated with each other, while no association between BRCA1 and p53 was found (data not shown).

**Table 3:** The Associations of P53, BRCAness Profile and Residual Tumor with 3-year Recurrence-Free Survival.

P53 and BRCAness*		3-Year		
		Recurrence-Free	Cumulative	
	Patients	Survival	Incidence	P value
	No. (%)	% (Stderr)	% (Stderr)	(Logrank)
**Overall**		60.2 (0.04)	30.8 (0.04)	
**By group**				
PARP				
Negative	74 (40.0)	67.5 (0.07)	24.7 (0.05)	0.14
Positive	111 (60.0)	56.5 (0.05)	36.2 (0.05)	
FANCD2				
Negative	105 (60.7)	66.9 (0.05)	27.5 (0.05)	0.15
Positive	68 (39.3)	55.5 (0.07)	35.8 (0.06)	
H2Ax				
Negative	70 (40.7)	59.0 (0.07)	30.9 (0.06)	0.76
Positive	102 (59.3)	64.2 (0.05)	30.8 (0.05)	
PTEN				
Negative	153 (88.9)	61.8 (0.05)	31.2 (0.04)	0.67
Positive	19 (11.1)	64.8 (0.06)	27.8 (0.11)	
ATM				
Negative	92 (53.8)	62.0 (0.06)	31.0 (0.05)	0.86
Positive	79 (46.2)	65.2 (0.06)	28.3 (0.05)	
BRCA1				
Negative	144 (86.7)	62.0 (0.05)	31.1 (0.04)	0.82
Positive	22 (13.3)	65.5 (0.12)	28.1 (0.10)	
P53				
Negative	81 (45.0)	64.9 (0.06)	27.4 (0.05)	0.54
Positive	99 (55.0)	58.1 (0.06)	34.7 (0.05)	
Residual Tumor				
Absent	59 (32.1)	58.1 (0.07)	37.6 (0.06)	0.61
Present	125 (67.9)	59.7 (0.05)	30.0 (0.04)	

*Negative is defined as no or weak staining; positive is defined as moderate or strong staining.

### Correlation of FANCD2 and PARP Expression with Clinical Outcome

**Figure 2:**
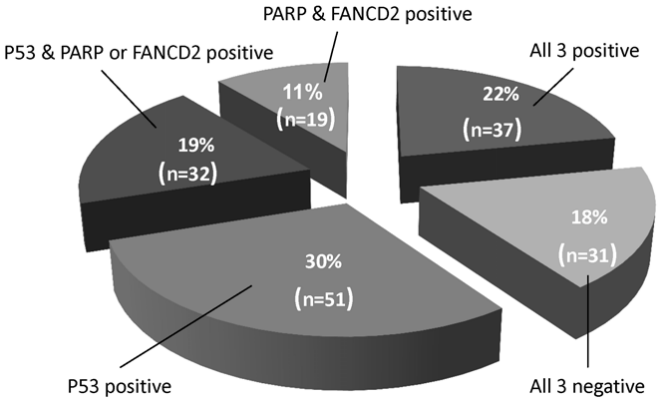
Distribution of five mutually exclusive groups of P53, PARP and FANCD2. Distribution of five mutually exclusive groups of P53, PARP, and FANCD2. 37 patients (22%) were positive for all three of the proteins.

**Figure 3:**
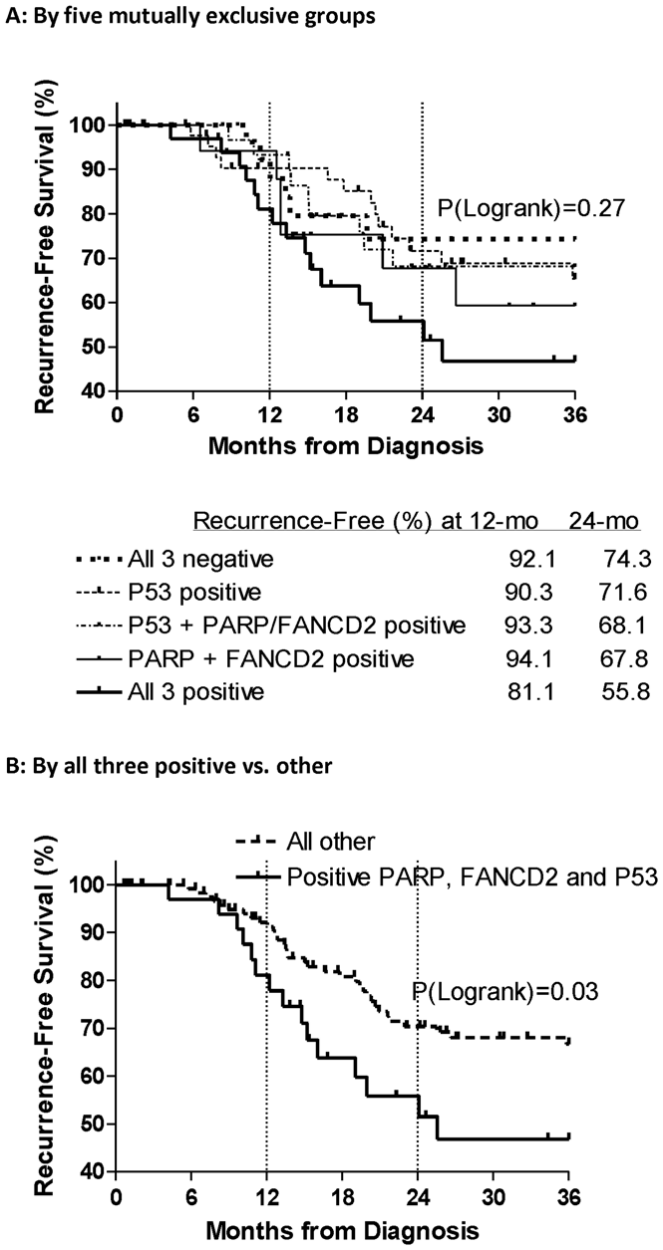
Kaplan-Meier Estimated 3-Year Recurrence-Free Survival Curves. Kaplan-Meier estimated 3-year recurrence-free survival curves. **A:** No difference in recurrence-free survival when each of the 5 mutually exclusive groups by expression of PARP, FANCD2, and P53 were examined independently. **B:** Patients positive for all three PARP, FANCD2, and P53 had lower recurrence-free survival compared to patients not positive for all three.

Patients were divided into five mutually exclusive groups based on their expression of PARP, FANCD2, and p53 (Figure 2). Samples stained negative for all three proteins in 31 patients (18%), positive for p53 alone in 51 patients (30%), and positive for FANCD2 and PARP only in 19 patients (11%). Over 40% of patients with positive PARP and/or FANCD2 had p53 protein overexpression, including 37 (22%) with overexpression of all 3 proteins, and 32 (19%) with p53 and PARP or FANCD2 overexpression. KM estimated 3-year recurrence-free survival curves for these mutually exclusive groups of p53, PARP and FANCD2 are displayed in Figure 3, and the impact of the BRCAness profile on recurrence-free survivorship is shown in Table 4. Differences in survival of more than 10% at 12-months and nearly 20% at 24-months was observed between the group with positive staining for all three proteins (p53, PARP and FANCD2) and the group with all three negative staining. The survival trended toward being shorter in all three positive groups compared to the other groups, although this was not statistically significant in this small sample size (Figure 3A). We further estimated 3 year recurrence-free survival curves between the patients with high levels of all three proteins (p53, PARP, FANCD2) and “other” patients as shown in Figure 3B. Patients with all high levels were more likely to have earlier recurrence within 3 years compared to all other patients (p = 0.03). At 12 months after diagnosis, the recurrence-free survival was 81% for patients with all positive levels of PARP, FANCD2 and p53 while the remaining patients had a recurrence-free survival rate of 92%. At 18 months, the recurrence-free survival was 64% for the group with all three positive proteins and 81% for all others, and the cumulative recurrence incidence rate was 16.5% for patients with all high levels of PARP, FANCD2 and p53 compared to 31.8% for the other patients. Patients with concomitant high levels of PARP, FANCD2 and p53 were twice as likely to have recurrence within 3 years, after adjustment for age and cancer status at the time of diagnosis (Table 4). The potential impact of residual tumor and other baseline clinical factors on early recurrence was accounted for using multivariate analysis. Notably, the presence of residual tumor was not associated with disease recurrence. However, patients with residual tumor after initial surgery tended to have positive PARP, FANCD2, and P53 expression in comparison with the group with no residual tumor (26% vs 14%, P = 0.07).

**Table 4:** High Levels of PARP, FANCD2 and P53 on Risk of 3-Year Recurrence.

		KM Recurrence-Free Survival (%)/				Cox Proportional Hazard	
Group	Patients	Cumulative Incidence (%)				Unadjusted	Adjusted*
	No. (%)	6-mo	12-mo	18-mo	24-mo	HR (95% CI)	HR (95% CI)
**Other**	133 (78.2)	99.2/0.8	92.1/7.1	80.8/16.5	70.4/24.5	1	1
**All Three Positive**	37 (21.8)	97.0/2.9	81.1/17.1	63.8/31.8	55.8/37.8	1.92 (1.05, 3.54)	1.88 (1.02, 3.49)

*Adjusted for age, cancer status (grade, histology type and stage) at diagnosis and the presence of residual tumor at surgery.

**Figure 4:**
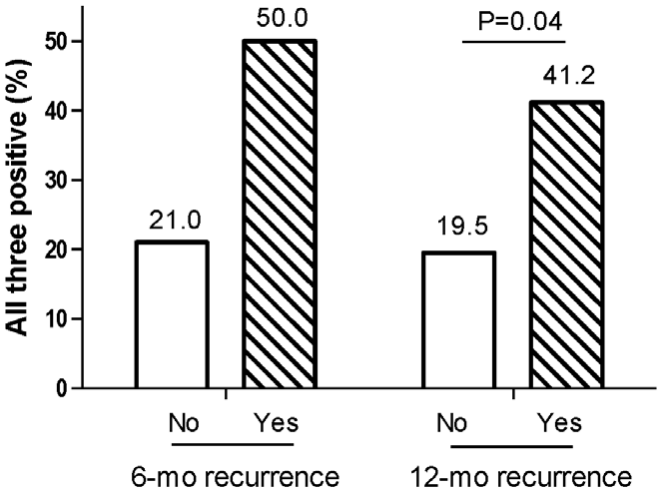
Association between Early Recurrence and Positive P53, PARP, and FANCD2. Association between positive P53, PARP, and FANCD2 in patients with and without early recurrence. Patients with early recurrence at both 6 and 12 months after diagnosis were much more likely to have high levels of all 3 proteins (50% at 6 months and 41.2% at 12 months).

In this study, we defined platinum resistant disease as disease recurring within 12 months of primary treatment completion, while recognizing that the more standard definition would mark platinum resistance as recurrence after 6 months. Therefore, Figure 4 explores the concurrent expression of PARP, FANCD2 and P53 in patients with and without 6-month and 12-month recurrence. Among the 4 patients who had recurrence within 6 months, 2 (50%) had all 3 positive, while 34 of 162 (21%) without recurrence had all 3 positive levels. Among the 17 patients who recurred within the first year of diagnosis, approximately 41% (7/17) had tumors with high levels of all 3 proteins, compared to 19% (29/149) with all 3 positive in no recurrence group (P = 0.04). Therefore, early recurrence, which is representative of platinum resistant disease, seems to be marked by concomitant high levels of PARP, FANCD2 and p53.

## Discussion

Recently, the role of DNA repair genes and proteins in ovarian cancer, the concept of BRCAness of ovarian cancers, as well as the mechanism of synthetic lethality has emerged as essential for the understanding of ovarian cancer pathogenesis and pathways to new treatments [7], [8]. As 20% of high grade serous sporadic ovarian carcinomas have BRCA1 and BRCA2 mutations, the role of HR in ovarian cancer has been well established [15], [16]. However, many hereditary and sporadic ovarian cancers do not possess BRCA mutations or altered BRCA expression and thus we reasoned that loss of other HR proteins may contribute to ovarian cancer. In fact, epigenetic silencing of FANCF via promoter methylation [17] and reduced levels of FANCD2 [18] have been previously detected in ovarian cancer. We further reasoned that overexpression of different HR proteins may account for acquired platinum-resistance and therefore can be used as a potential screen to (i) identify patients at a higher risk for recurrence and (ii) identify new therapeutic targets. To measure the BRCAness (i.e. the HR) profile of sporadic ovarian cancers, we used immunocytochemistry (ICH) to examine protein expression of DNA damage response proteins FANCD2, BRCA1, PARP, H2AX, and ATM, as well as PTEN and p53, tumor suppressor genes that also have a function in maintaining genomic stability by engaging in DNA repair [19]–[25] in a panel of sporadic ovarian carcinomas.

To our knowledge, there has been no previous systematic analysis of ATM and H2AX expression in ovarian cancers. Here we found that the proteins were expressed in 46% and 59% of our samples respectively, which may indicate their potentially compensatory expression in HR deficient cells.

Supporting the role of p53 and PTEN as tumor suppressors, the majority of ovarian carcinomas tested was positive for p53 (55%), as the antibody detects both wildtype and mutated forms of p53, and negative for PTEN expression (89%). These results are similar to other studies with advanced cancers [21]–[22].

Interestingly, we found that 86.7% of the cases had negative BRCA1 ICH. Most of the prior studies on BRCA1 deficiency in sporadic ovarian cancer have focused on mutational analysis, methylation studies, and gene expression, with only a few immunocytochemical studies. A recent report by Skytte et al. revealed negative BRCA ICH in only 20% of ovarian cancers [26]. These differences may be explained by the use of different antibodies to detect the BRCA protein [27]. In fact, in a comparative study of 84 cases with sporadic breast cancer with four different anti-BRCA antibodies, Al-Mulla et al. detected BRCA1 protein expression loss in 83% of the cases using the same BRCA antibody AB-1, against the N-terminus epitope [28]. In the NCIC study which correlated BRCA1 protein expression by ICH with corresponding clinical data, 251 ovarian cancer samples were analyzed using a mouse monoclonal BRCA1 antibody (MS110, Calbiochem, Germany) and 65% of the tumors showed no staining or very mild staining [29]. The antibody used in our study was different from the antibodies used in the prior studies. In addition, because of the small sample size in our study and similarly in the NCIC study, our scoring grouped the staining results of four categories to negative (no or weak) and positive (moderate or strong) based on staining intensity. It therefore remains necessary for future studies to clearly outline the guidelines for BRCA1 staining and scoring in studies of this marker. Nevertheless, the loss of BRCA1 in the majority of tumors in our study coupled with the increased expression of PARP in most of the cancers (discussed below) suggests that PARP upregulation may be a compensatory mechanism by cancer cells to withstand platinum-therapy-induced death.

Our results with PARP are also consistent with previous studies. We found high expression of PARP in 60% of specimens, similar to prior reports suggesting positive PARP protein staining in 76% of ovarian carcinomas [26]. Moderate FANCD2 staining was found in most (85%) stage I serous carcinomas of the ovary [Pejovic, unpublished data], while in this series we find that 40% of advanced serous carcinomas overexpress FANCD2. Thus, overexpression of FANCD2 in addition to PARP may increase the capacity of cells to repair DNA double- and single-strand breaks contributing to platinum resistance.

Synergy among PARP, FANCD2 and p53 protein expression is one of the striking results in our study. Tumors with high expression of PARP and/or FANCD2 were more likely to show overexpression of p53. The protein profile defined by high expression of all three proteins PARP, FANCD2, and p53 is associated with a high risk of recurrence. Not only was recurrence rate at three years twice as high in this group, but it is particularly noteworthy that over half of patients recurring within the first 12 months after primary treatment (platinum resistant disease) had significantly elevated expression of these three proteins. This subgroup of patients with particularly difficult to treat ovarian cancer has not been previously identified by protein or other molecular profiling. Thus, these results allow both identification of women at higher risk for relapse and suggest potential therapeutic targets. Recently, promising results were reported in a phase II clinical trial of PARP inhibitor BSI-201 (iniparib) in combination with gemcitabine and carboplatin in 19 patients with platinum resistant ovarian cancer [30].

Our study has several limitations. First, though we examined 186 patients, this is still a limited sample size for all clinical correlations examined. Second, patients who are BRCA1 and BRCA2 mutation carriers have not been identified in this study. The strict guidelines for prognostic studies using immunocytochemistry using standardized criteria are still being developed. Also we examined only a subset of DNA repair proteins, while inclusion of other proteins involved in the HR such as ATR, CHK2 or RAD51, and functional studies of RAD51 foci formation to evaluate the sensitivity of these cells to PARP (and FANCD2) inhibition are currently underway.

Finally, while we believe that the most important results of this study are (i) identification of a BRCAness profile that is not associated with BRCA1 and (ii) association of this triple-positive ovarian cancer (FANCD2+/PARP+/P53+) with very early recurrence of ovarian cancer and therefore platinum resistance. These patients may benefit from dual inhibition of FANCD2 and PARP and/or small molecules targeting mutated p53. In fact, inhibition of the FA/BRCA pathway in sporadic cancers by different compounds including circumin, has been shown to sensitize cancers to platinum agents [31]. Thus, combined treatment of FA/BRCA pathway inhibitors and PARP inhibitors already in clinical trials may be an effective treatment for relapsed patients that have been identified to overexpress FANCD2 and PARP.
